# Hardy personality and burnout syndrome among nursing students in three Brazilian universities—an analytic study

**DOI:** 10.1186/1472-6955-13-9

**Published:** 2014-03-30

**Authors:** Rodrigo Marques da Silva, Carolina Tonini Goulart, Luis Felipe Dias Lopes, Patrícia Maria Serrano, Ana Lucia Siqueira Costa, Laura de Azevedo Guido

**Affiliations:** 1 Department of Nursing, Federal University of Santa Maria, 78 Fioravante Antonio Spiazzi St., Zip: 97095-180 Santa Maria, Rio Grande do Sul, Brazil; 2 Department of Administration, Federal University of Santa Maria, Santa Maria, Rio Grande do Sul, Brazil; 3 Department of Nursing, Paulista University (UNIP), Sorocaba, São Paulo, Brazil; 4 Medical-Surgical Nursing Department, Nursing School of the University of São Paulo (EEUSP), Sao Paulo, São Paulo, Brazil

**Keywords:** Burnout, Nursing, Nursing education, Students, Stress, Psychological adaptation, Professional burnout

## Abstract

**Background:**

Nursing students may exhibit the characteristics of resistance to stress, such as hardiness, which can reduce the risk of burnout. However, we found only one published study about these phenomena among nursing students. Thus, we investigated the association between hardiness and burnout in such students.

**Methods:**

An analytic, cross-sectional study was conducted among 570 nursing students from three Brazilian universities. Data were collected relating to sociodemographic characteristics, hardiness, and burnout, which we analyzed using inferential statistics.

**Results:**

We observed that 64.04% of nursing students in the sample had a high level of emotional exhaustion, 35.79% had a high level of cynicism, and 87.72% had a low level of professional efficacy: these are dimensions of burnout. We also found that 48.77% had a high level of control, 61.40% a high level of commitment, and 35.44% a high level of challenge: these are dimensions of hardiness. Only 24.74% of the students experienced burnout, and 21.93% met the criteria for a hardy personality. There was a statistically significant difference between the frequency of hardiness and burnout (*p* = 0.033), with 68.00% of hardy students not exhibiting burnout.

**Conclusions:**

Although nursing students live with educational stressors, burnout was not preponderant in our sample students; this may be linked to hardiness. Thus, given its benefits to student life and health, we recommend the development of strategies to promote hardiness among nursing students.

## Background

Undergraduate nursing education is a very important stage in a nurse’s career. During their undergraduate education, students experience academic demands—tests, theoretical and practical course work, research activities, and aspects of professional practice, such as contact with health professionals and patients—as well as the practical matters of providing health services. Thus, students face situations related to their profession and academic development that may be assessed as stressors [1].

Research in various countries has identified a number of stressors that are experienced by university students, including academic activities experienced in the first 12 months of college. For nursing students, these stressors include the following: traumatic experiences with death; responsibility in caring for other people; requirements regarding performance and interpersonal relationships; feeling unprepared to perform functions that need to be exercised in practical classes; and adaptation to academic requirements [2-4].

Research in Brazil has indicated that intimate contact with patient pain, patient suffering, care for terminal patients, and physical intimacy with patients are all common stressors in the nursing profession. In an academic context, students may regard examination periods, the transition from high school to college, and tasks outside their class requirements as stressors [5]. In a study of nursing students in São Paulo, the most highly rated stressors were found to be making mistakes at work, harming patients, and receiving contradictory instructions [6]. Stress occurs when internal or external sources produce a situation that is interpreted as exceeding a person’s adaptive resources, and this has possible repercussions on the physical and mental health of college students [7].

A statistically significant correlation between stress and burnout syndrome has been reported [8,9]. This syndrome occurs when individuals are unsuccessful in using coping strategies; stressors remain, which may lead to chronic stress [10]. The features of burnout among students are as follows: emotional exhaustion, which is characterized by feelings of exhaustion owing to study requirements; cynicism, which entails a cynical, detached attitude toward study; and professional inefficacy, which is marked by the perception of oneself as incompetent [10]. Although most students live with academic and professional stressors, it has been found that stress tends to be low among undergraduate students [11] and moderate among graduate students [12].

Because it is a personality characteristic that provides resistance to stress, a hardy personality has been proposed as an explanation for the occurrence of low stress among different populations [13]. Hardy people have three common characteristics: (a) they believe they can control events in their lives (control); (b) they are able to consider themselves as fully engaged in their daily activities (commitment); and (c) they are capable of interpreting problems as exciting challenges to personal growth (challenge) [13]. As a result of their experiences, such individuals are able to adapt over time, and this appears to be related to better physical and mental health [13].

Thus, although stress can lead to burnout, hardiness provides resistance to stress, and it can protect students against burnout. We conducted a literature review on PubMed to scan the scientific literature about burnout and hardiness among nursing students. Our search identified a 1997 study that investigated these attributes among a sample of oncology nurses [14]. That study appeared to be the only one to examine both hardiness and burnout in nursing students, and only a few investigations have examined burnout in this population.

It is important to study burnout among nursing students because this condition has been found to be related to decreased academic performance, which can influence the quality of care in the nurses’ professional life and expose patients to care-related risks. Burnout may also cause nurses to leave their careers during the first years of work, which can lead to nursing staff overload and high levels of absenteeism [15,16]. Conversely, hardiness provides a buffer to stress: it has a protective effect against burnout and the negative outcomes of burnout during nursing education and practice.

Based on our examination of the literature, we posed three research questions: Does burnout occur among hardy individuals? Does hardiness occur among the nursing students in the colleges in our study area? What is the association between hardiness and burnout? The present study investigated the association between hardiness and burnout among nursing students at three Brazilian universities. We hypothesized that hardiness provides resistance to stress and thus reduces burnout among nursing students.

## Methods

This was an analytic, cross-sectional study. It was conducted at three Brazilian higher education institutions, two in the Southeast Region and one in the South Region.

### Inclusion and exclusion criteria

We included undergraduate students in three nursing degree programs, enrolled from the first to eighth semesters of their respective degree programs. All the students were over 18 years of age. We excluded the following students: those who were not enrolled in courses specifically related to nursing care; those who could not conclude the curriculum because they had exceeded the time limit at their college; those who were not present on the day when data were collected; and those who were studying abroad.

We collected the data between April 2011 and March 2012 by approaching the students during classroom time. Prior arrangements to do so in scheduled classes were made with the teaching staff.

### Study population

The initial potential sample consisted of 732 nursing students enrolled in three nursing colleges. However, the following were excluded: 14 students not enrolled in courses specifically related to nursing care; three students unable to conclude the curriculum because they had exceeded the college time limit; 91 students not present in class when the data were collected; three students studying abroad; four students aged under 18 years; 34 students who failed to return the research instruments; and four students participating in the project as researchers. Thus, 153 students were excluded. In addition, eight students refused to participate in the study, and one did not respond to all the items on the Hardiness Scale (HS). Therefore, 570 nursing students comprised the sample of this study.

### Data collection

We collected the data through the following self-report instruments: a form to collect the sociodemographic characteristics of the students; the Maslach Burnout Inventory-Student Survey (MBI-SS); and the Hardiness Scale. We distributed these instruments to the participants who voluntarily agreed to participate in the study after signing a form signaling their free, informed consent.

The sociodemographic characteristics included the following variables: date of birth, number of children, sex, marital status, and the people with whom the student lived.

The MBI-SS was translated and adapted for Brazil in 2006 [17]. This instrument is designed to assess burnout syndrome among university students, i.e., how they experience their education, according to three conceptual subscales: emotional exhaustion, cynicism, and professional efficacy [17]. It is a self-report questionnaire composed of 15 items, which are rated on a seven-point Likert scale: 0, never; 1, at least once a year; 2, less than a few times a month; 3 a few times a month; 4, once a week; 5, a few times a week; and 6, every day. The items are distributed among the subscales as follows: emotional exhaustion (items 1, 4, 6, 8, and 12); cynicism (items: 2, 9, 10, and 14); and professional efficacy (items 3, 5, 7, 11, 13, and 15) [17].

The HS was adapted from the 2009 Brazilian version [18], which was validated in 2012 [19]. It is composed of 30 items, which are rated on a four-point Likert scale: 0, not true; 1, somewhat true; 2, almost completely true; and 3, completely true. The items are distributed in three domains: control (items 2, 3, 8, 9, 12, 15, 18, 20, 25, and 29); commitment (items 1, 6, 7, 11, 16, 17, 22, 27, 28, and 30); and challenge (items 4, 5, 10, 13, 14, 19, 21, 23, 24, and 26). The ratings of the following items were assessed before the domain scores were totaled: 3, 4, 5, 6, 8, 13, 16, 18, 19, 20, 22, 23, 25, 28, and 30 [18].

### Data analysis

The analyses of the instruments were conducted using standardized scores (*Sp*_*i*_), which were calculated for each subscale of MBI-SS and each domain of the HS. To do this, we totaled the responses, and the resulting figure was subtracted from the sum of the minimum possible values for each subscale or domain. That sum was divided by the difference between the maximum total value and the sum of the minimum possible values for each subscale or domain.

Using the final scores, we classified the levels of emotional exhaustion, cynicism, and professional efficacy on the MBI-SS as follows: students with scores of 50% or less were classified as low on the respective subscale; those with scores above 50% were classified as high. Students that scored high on emotional exhaustion, high on cynicism, and low on professional efficacy were considered to be experiencing burnout [17].

For HS analysis, the classifications of control, commitment, and challenge levels were performed the same way: students with scores of 50% or less were classified as low in that domain; those with above 50% were classified as high. Hence, students that had concomitantly high control, high commitment, and high challenge were considered to have a hardy personality [13].

After collection, the data were entered and stored on an Excel spreadsheet (Microsoft Office Package 2007) to be analyzed with the Statistical Analysis System (SAS) (Version 9.01), a software developed by SAS Institute, and Statistica (Version 9.01) which was created by StatSoft. The qualitative variables were presented as absolute (n) and relative (%) values, and the quantitative variables were presented as descriptive statistics, such as the minimum and maximum values, average (i.e., mean), and standard deviation. Fischer's exact probability test was used to test the association between the frequency of burnout and hardiness. *p* < 0.05 was considered statistically significant with a confidence interval of 95%. Cronbach’s alpha coefficient was used to analyze the internal consistency of the instruments.

### Ethical aspects

This study was part of the project named Stress, Coping, Burnout, Depressive Symptoms and Hardiness in Students and Teachers of Nursing, which was approved by the Ethics Research Committee at the University in the South of Brazil (protocol No. 0380.0.243.000-10). We requested the committee for an amendment to expand the data collection to other schools, and we obtained approval for this. To meet the guidelines and standards of the Brazilian National Health Council, established for studies involving human subjects, the participants voluntarily consented to participate in the study by signing two copies of free, informed consent forms (one for the participant and the other for the researcher) after being informed of the objectives of the study.

## Results

The analysis of internal consistency of the items that compose the MBI-SS gave a Cronbach’s alpha of 0.596. For the MBI-SS subscales, this coefficient was 0.769 for emotional exhaustion, 0.623 for cynicism, and 0.612 for professional efficacy. Cronbach’s alpha was 0.781 for all 30 items of the HS: it was 0.643 for control, 0.644 for challenge, and 0.643 for commitment. These values are sufficient to attest to the instrument’s satisfactory internal reliability [20].

With respect to the sociodemographic characteristics, the sample group was predominantly female (84.21%), between the ages of 20 and 24 years old (47.37%), single (74.39%), without children (81.05%), and lived with family (75.57%). Descriptive statistics for the MBI-SS and HS among the nursing students are presented in Table 1.

**Table 1:** Descriptive statistics for the MBI-SS and HS among nursing students, Brazil, 2013

**Instruments**	**Descriptive statistics**			
**MBI-SS**	**Mean**	SD*	**Minimum**	**Maximum**
15 items	2.51	0.79	0.40	4.87
Emotional exhaustion	3.57	1.31	0.00	6.00
Cynicism	1.78	1.29	0.00	5.75
Professional efficacy	2.12	0.82	0.83	5.67
**HS**				
30 items	2.05	0.33	1.12	3.00
Commitment	2.15	0.42	1.00	3.00
Control	2.06	0.38	1.00	3.00
Challenge	1.94	0.41	1.00	3.00

*Standard deviation.

We observed that 64.04%% of students had high levels of emotional exhaustion, 35.79% high levels of cynicism, and 87.72% low levels of professional efficacy. This indicates that some students were feeling emotionally exhausted (possibly because of academic requirements); they were treating people in a detached way and feeling incompetent as students given the academic demands. The analyses of the subscales revealed that 24.74% of the participants were experiencing burnout, i.e., they were living with chronic stress, possibly caused by the academic environment.

With regard to hardiness, we found 48.77% of students with a high level of control, 61.40% with a high level of commitment, and 35.44% with a high level of challenge. These results indicate that some of the nursing students were trying to control their situation rather than being passive and powerless about their situation. They also appeared to remain involved with people and situations and to interpret stressors as opportunities to learn. When we analyzed these domains, 21.93% of such students exhibited the characteristics of a hardy personality.

There was a statistically significant difference (*p* = 0.033) between the frequency of burnout and hardy personality among the nursing students (Table 2). Burnout frequencies thus differed significantly in relation to the frequency of hardiness.

**Table 2:** Comparison between the frequency of burnout and hardiness among nursing students, Brazil, 2013

	**With hardiness n (%)**	**No hardiness n (%)**	**Total**	***p *** **value**
**With burnout,**	40	101	141	
**n (%)**	(7.02%)	(17.72%)	(24.74%)	
**No burnout,**	*85*	344	429	
**n (%)**	*(14.91%)*	(60.35%)	(75.26%)	
**Total,**	125	445	570	0.033
**n (%)**	(21.93%)	(78.07%)	(100.00%)	

After analyzing the above frequencies, we verified that of the 125 (100%) students with a hardy personality, 68% (n = 85) did not experience burnout. Therefore, a hardy personality appears to protect students against burnout and, presumably, its negative outcomes.

## Discussion

When students undergo the teaching and learning process, they may perceive different situations related to theoretical and practical activities as being stressful. Thus, it is possible that they use coping strategies to minimize the effects of stress. However, when these strategies are not used or are ineffective for a given stressor, stress remains and may lead the students to experience burnout.

Our results indicate that 64.04% of the nursing students in the sample had high levels of emotional exhaustion, 35.79% high levels of cynicism, and 87.72% low levels of professional efficacy. A study of 545 medical students in Minnesota (USA) found that 34.7% had high emotional exhaustion, 25.8% had high cynicism, and 30.8% experienced feelings of low professional efficacy [21]. Another study conducted among medical students in Sergipe (Brazil) found that 62.60% had high emotional exhaustion, 47.40% high cynicism, and 60.20% low professional efficacy [22]. These investigations indicate that there is a preponderance of students with high emotional exhaustion and low professional efficacy. This observation deserves attention because although burnout is a tridimensional syndrome, emotional exhaustion is thought to be an initial feature of burnout syndrome and is mainly experienced by individuals as mental depletion [10,17]. In that regard, a study of 1,702 nursing students, who were observed for 3 years in a longitudinal study [23], found that the level of emotional exhaustion among students increased with time; there were significant correlations between emotional exhaustion and high levels of depression and low levels of life satisfaction [23]. With respect to low professional efficacy, feelings of being incompetent as a student have been linked to the desire to leave undergraduate education and to depression among students [17,24,25]. Given the negative effects of burnout on students’ health, these findings thus highlight the importance of implementing strategies to minimize emotional exhaustion and feelings of low professional efficacy.

When the MBI-SS subscales were analyzed, we observed that 24.74% of the students were experiencing burnout. In a study of nursing students in São Paulo, none of the students exhibited burnout [26]. Research of Brazilian students in other courses found that 17% of 235 dental students [27] and 10.3% of 369 medical students exhibited burnout [22]. In the United States, research has reported that 48.6% (n = 4,287) of medical students in USA [28] and 45% (n = 545) of medical students in Minnesota [21] experienced burnout. These results suggest that the frequency of burnout may be higher among students in other degree programs than in nursing. This may be related to different academic contexts and the functions performed in each undergraduate course of study.

According to the authors of an investigation of dental students [27], financial factors related to the course and the specific nature of the work, such as activities restricted to the oral cavity, characterize dentistry as a stressful profession. This may explain the relatively large number of dental students with burnout. Studies of medical students [21,22,28] have attributed the high prevalence of burnout to insecurity regarding the skills required to become a doctor and feelings of discomfort linked to the course activities. Furthermore, students that are involved in clinical activities, such as interaction with patients, have feelings of uncertainty and increased responsibility, which are notable predictors of burnout [22]. Hence, one study with American nursing students [26] pointed out the necessity to improve the teaching-learning environment and organization of clinical activities as well as broadening the students’ experiences.

Nursing students experience similar situations to the above students as well as those that are specific to nursing, such as managing health services, planning and implementing care, and the necessity of exercising leadership in health-care teams. However, given their lower prevalence of burnout, nursing students appear to interpret and deal with stressors in a different way than students in other disciplines.

In this study, we found that 48.77% of the students experienced a high level of control, 61.40% a high level of commitment, and 35.44% a high level of challenge. When the HS domains were analyzed, we found that 125 students had a hardy personality. Of these hardy students, 68.00% did not experience burnout (*p* = 0.033). Thus, the characteristics of the nursing students in our sample tended to be the belief in being able to control events related to the teaching and learning process, the commitment to academic activities, and the ability to interpret potentially stressful situations in the educational process as a challenge, which signifies hardiness. Consequently, these individuals appear to suffer less from stress.

Various studies have been conducted to examine the influence of a hardy personality on the occurrence of different phenomena related to stress. In research with college students in Ohio (USA), the results indicated that hardy students are less likely to suffer depression when exposed to the stressors of personal and academic situations [29]. An investigation of students in California (USA) found that a hardy personality was the strongest predictor of academic performance when compared with other variables, such as well-being, academic attitudes, and life satisfaction [30]. In a study of Iranian students, there was a statistically significant, negative correlation between hardiness and mental disorders [31]. In view of the benefits of hardiness to health, researchers have applied a strategy to promote hardiness, based on theoretical training, to increase the academic performance of college students in California [32]. It may also be advantageous to encourage hardiness among nursing students to promote a satisfying educational process. This would help develop qualified professionals to provide nursing care and reduce the health risk among future nurses.

## Conclusions

We did not find a preponderance of burnout syndrome among the nursing students in this study. However, some students had high emotional exhaustion, which is an initial feature of the syndrome, and this warrants attention to avoid an increase in the number of nursing students with burnout. The absence of burnout in 68.00% of the hardy population and the statistically significant difference in the association among the variables reinforce the assertion that hardiness is a characteristic that promotes health and reduces disease, which was the premise of this research. We hypothesized that hardiness protects students from succumbing to burnout, and our results support this hypothesis. Because burnout has negative effects on the academic performance of nursing students and, in the long term, on the quality of nursing care, we recommend that interventions to promote hardiness be implemented for nursing students to avoid or reduce the occurrence of burnout.

There are some difficulties when comparing results about hardiness reported by national and international studies. This is because they sometimes equate hardiness with other phenomena and do not report the prevalence of the hardy personality or its domains across different populations.

## Competing interests

The authors declare that they have no competing interests.

## Authors’ contributions

RMS participated in drafting the manuscript, the interpretation of data and the critical review of the paper’s intellectual content; CTG participated in drafting the manuscript and the critical review of its intellectual content; LFDL undertook the statistical analysis and interpretation of data; PMS drafted the manuscript and participated in the critical review of its intellectual content; ALSC assisted in drafting the manuscript and participated in the critical review of its intellectual content; LAG carried out the design and acquisition of data, drafting the manuscript, and the critical review of its intellectual content. All the authors have given their final approval of the version to be published.

## Pre-publication history

The pre-publication history for this paper can be accessed here:

http://www.biomedcentral.com/1472-6955/13/9/prepub
